# Differentiating the clinical and computed tomography imaging features of mixed epithelial and stromal tumors of the kidney to establish a treatment plan

**DOI:** 10.1002/acm2.13486

**Published:** 2021-12-03

**Authors:** Juan Chen, Hui Liu, Mengsi Li, Wenguang Liu, Ismail Bilal Masokano, Yigang Pei, Wenzheng Li

**Affiliations:** ^1^ Department of Radiology Xiangya Hospital Central South University Changsha Hunan China; ^2^ Postdoctoral Fellow Xiangya Hospital Central South University Changsha Hunan China

**Keywords:** classification, clinical data, mixed epithelial and stromal tumor of the kidney, multidetector computed tomography features, patient management

## Abstract

**Objective:**

To differentiate the clinical features and computed tomography imaging features in the two types of mixed epithelial and stromal tumor of the kidney (MESTK) and to establish a treatment plan for the MESTK types.

**Methods:**

Seventeen patients who underwent multidetector computed tomography (MDCT) before surgery and had a pathological diagnosis of MESTK were enrolled. Their clinical information (R.E.N.A.L. nephrometry score (R.E.N.A.L.‐NS), radical nephrectomy (RN), partial nephrectomy (PN), etc.) were collected. The radiological features included renal sinus fat invagination (SFI), maximal diameter (MD), capsule and septa of the tumor, etc., were also analyzed. They were divided into two types according to the MD_solid_/MD_tumor_ ratio (solid type with >60%; cystic type with ≤60%). An independent‐sample *t*‐test and Fisher exact test were used to assess the differences between the two groups.

**Results:**

MESTKs demonstrated a variable multi‐septate cystic and solid components with a delayed enhancement. There were nine patients for solid type and eight patients for cystic type. Compared with solid type, the lesions in cystic type have larger MD (81.00 ± 37.91 vs. 41.22 ± 24.19, *p* = 0.020), higher R.E.N.A.L.‐NS (10.03 ± 0.50 vs. 8.95 ± 1.26, *p* < 0.001), higher RN (75.00% vs. 22.22%, *p* = 0.015), larger SFI (87.5% vs. 33.3%, *p* = 0.05), more septa (100% vs. 0%, *p* < 0.001), and more capsule (100% vs. 11.1%, *p* < 0.001).

**Conclusion:**

Cystic type MESTK has more hazardous features (such as larger MD, higher R.E.N.A.L.‐NS, more RN, greater SFI, multiple septa) compared with solid type, suggesting that RN is more suitable for cystic type and PN for solid type.

## 1 INTRODUCTION

Mixed epithelial and stromal tumor of the kidney (MESTK), first identified by Michal and Syrucek,^1^ is a rare benign entity with a prevalence of 0.2%–1.6% of all renal tumors.^2^, ^3^ Histopathologically, MESTK is composed of epithelial and stromal components.^4^ It appears as a cystic renal mass with varying proportions of solid components on imaging. The WHO grouped the lesion into the mixed epithelial and stromal tumor family in 2016.^5^ In clinical practice, it is easy to misdiagnose MESTK as a malignant tumor, such as cystic renal cell carcinoma (CRCC).^6^, ^7^ Therefore, accurate preoperative identification of MESTK is necessary for clinicians to avoid extensive surgery, especially radical nephrectomy (RN). However, the accurate preoperative features of indeterminate MESTK remains a challenge for clinicians.

Although aspiration biopsy may be an effective method for preoperative identification of indeterminate renal tumors,^2^, ^8^ it is not recommended for cystic renal masses and is limited by its invasive nature, the relatively high misdiagnosis rate due to size limitations.^8^ Computed tomography (CT), as a non‐invasive imaging method widely used in the abdomen, could provide valuable diagnostic information to assist clinicians and radiologists in making a reasonable diagnostic decision when an indeterminate renal tumor is encountered. Although some imaging features has been reported for MESTK, only limited CT radiological features of MESTK have been presented for case reports and series.^6^, ^9^, ^10^ The classic imaging appearance of MESTK is that of a well‐circumscribed, multi‐septate cystic and solid mass with delayed enhancement.^11^, ^12^, ^13^ To the best of our knowledge, the largest radiological study on MESTK described the CT imaging features no more than eight cases.^7^, ^14^

Most of MESTK tumors are Bosniak category III or IV or solid lesions.^13^, ^15^ Therefore, surgical excision (partial nephrectomy (PN) and RN) is the most common treatment for patients with MESTK. PN should be recommended to preserve the nephron as much as possible in most MESTK patients because it is a benign entity. Therefore, an accurate evaluation of MESTK is vital for preoperative treatment planning. Furthermore, it is still unclear whether the imaging features and clinical data can help make a suitable treatment plan for the patients with MESTK.

In our study, the patients with MESTK were divided into two types based on the proportion of MD_solid_/MD_tumor_ in the maximal slice (solid type: the ratio >60%; cystic type: the percentage ≤60%). Our purpose is to analyze the clinical and radiologic features of MESTK, with emphasis on evaluating the differences in clinical and CT imaging features and proposing a patient management plan for the two types.

## 2 MATERIAL AND METHODS

### 2.1 Patients

Ethical approval was obtained from Institutional Review Board of the hospital. This retrospective study was performed in accordance with the provisions of the Declaration of Helsinki. The requirement for informed consent was exempted by the Institutional Review Board of the hospital, as all data were analyzed anonymously (Approved number 2018111101). From June 2011 to November 2020, 17 patients pathologically diagnosed with MESTK (13 women and four men, the median age was 45.12 ± 10.77 years (range from 21 to 61 years)), who underwent preoperative multidetector CT (MDCT) within 15 days before the surgical procedure were recruited. The clinical information (the follow‐up time, gender, age, clinical symptoms, menstrual status, surgery methods (PN and RN), and R.E.N.A.L. nephrometry score (R.E.N.A.L.‐NS)) were collected (R: radius (tumor size as maximal diameter (MD)); E: exophytic/endophytic properties of the tumor; N: nearness of tumor deepest portion to the collecting system or sinus; A: anterior (a)/posterior (p) descriptor; L: location relative to the polar line).^16^ For R.E.N.A.L‐NS, the range of 4–6, 7–9, and 10–12 were deemed low, moderate, and high complexity lesions, respectively. The beginning and end of the follow‐up period was the time the patient had surgery and the latest MDCT or ultrasound examination in our hospital.

### 2.2 MDCT techniques

All patients carried out plain and contrast enhancement MDCT scan in our hospital (128 slices, Somatom Definition, Siemens Healthineers, Erlangen, Germany; 320‐slice, Toshiba Aquilion ONE, Canon Medical Systems, Otawara, Tochigi, Japan). The scanning parameters were as follows: slice thickness of 1 mm, slice gap of 0 mm, the pitch of 1.2, 100 kVp and 200 mA for Somatom Definition and Aquilion ONE. For enhanced MDCT, a non‐ionic iodinated contrast agent (iopromide, Ultravist; Schering AG, Berlin, Germany) was used at a rate of 3.5–4 ml/s (1.5 ml/kg, 80–100 ml). An unenhanced scan was obtained before contrast agent injection and then the corticomedullary phase (CMP) (30 s), the nephrographic phase (NP) (70 s), and the excretion phase (EP) (3–5 min) were obtained for each subject after injection of the contrast agent.

### 2.3 Imaging analysis

All MDCT images were transferred into the imaging workstation (Advantage Workstation 4.4, GE Healthcare, Buc, France) and image post‐processing (sagittal and coronal images) was performed. Two experienced radiologists (reviewers 1 and 2 with 5 and 7 years of clinical experience in kidney MDCT, respectively) assessed the imaging features without knowing the clinical and pathological information, including the MD, shape, location, calcification, septa state, mural nodule, capsule of the tumor, renal sinus fat invagination (SFI), and the enhancement degree and pattern. SFI is defined as the direct contact of the tumor with the renal sinus stroma or fat cell,^17^ which appears as an invasion of the fat tissue of the renal sinus on MDCT. But it is not considered invasive if the tumor impinges on (but is separated from the fat by a connective tissue layer) the perinephric or renal sinus fat.^18^ Enhancement patterns were evaluated and analyzed with the CT attenuation for each phase for all MESTK. A region of interest (ROI) was drawn on the solid component of the tumor (size: 40–60 mm^2^) on EP images and copied to plain, CMP, and NP images of the same slice, avoiding the cystic and calcified parts in the tumor. For each subject, the above measurements were carried out two more times on different occasions within a weekend. The average CT attenuation was calculated for each phase to obtain the enhancement pattern (wash out or delayed enhancement). Gradual enhancement pattern was considered present when the tumor attenuation in the NP was at least 20 Hounsfield units (HU) greater than that in the CMP,^19^ while gradual washout pattern was defined when the CT value of the subsequent phase was reduced to less than 20 HU.^20^ The degree of enhancement was defined as the difference between the attenuation value of the unenhanced scan and the CMP. A difference higher than 50 HU was classified as marked, between 20 and 50 HU as moderate, and less than 20 HU as weak enhancement.^6^

**FIGURE 1:**
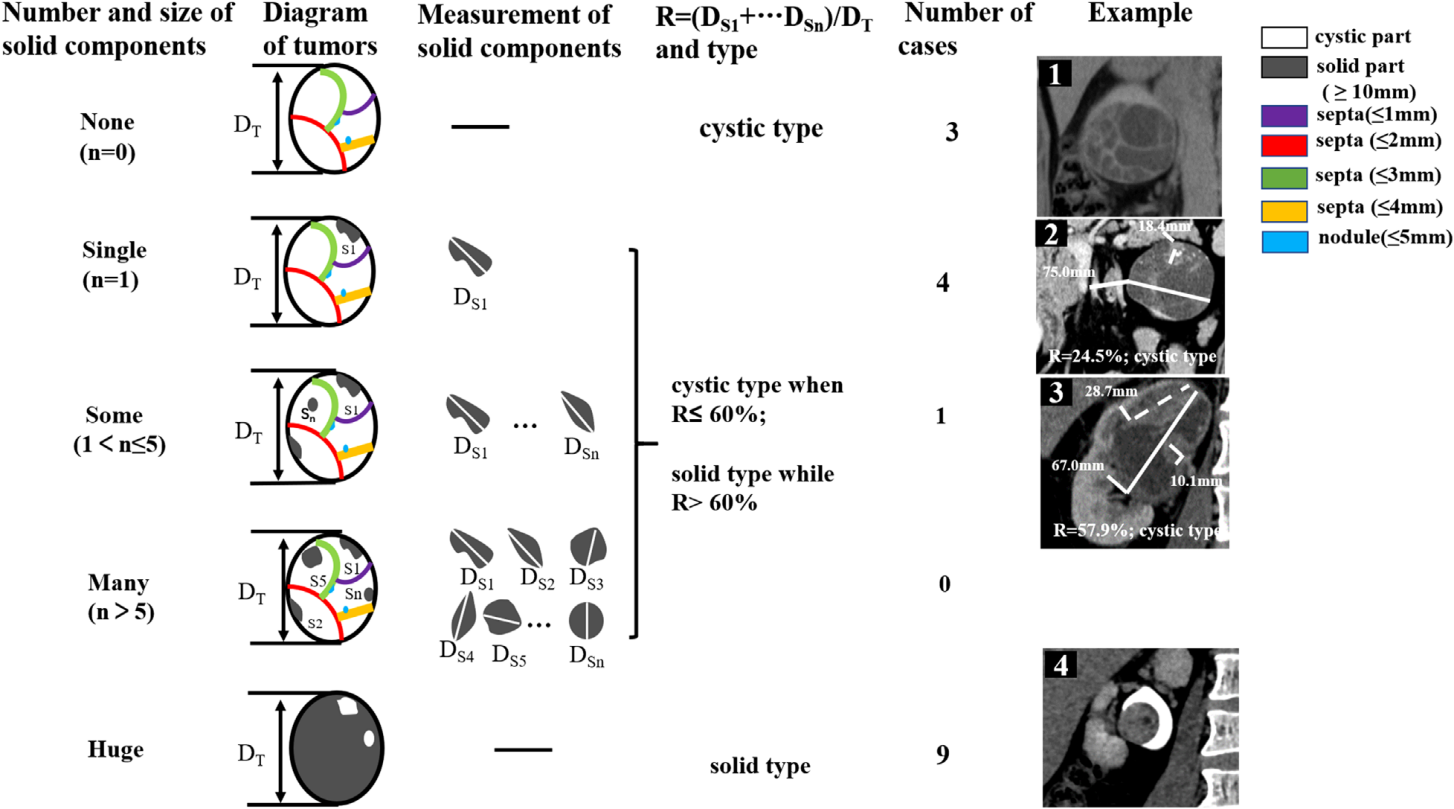
The schematic diagram of mixed epithelial and stromal tumor of the kidneys (MESTKs) classified into solid type and cystic type based on the proportion of solid components. A threshold value of 25% is defined in the Bosniak classification (version 2019) of renal cystic masses with solid components, which is the volume proportion of solid components in the mass and the volume is relative to the respective maximal diameter (MD) (*V* = (4/3)π*R* ^3^ and *R* = MD/2). So, the MD was used to replace the corresponding volume in our study (63% was obtained by the above formula and should be the threshold for the MD ratio of solid part). Note that 60% was used to the threshold of MD proportion of solid components due to the difficulty of measuring septa and nodule less than 5 mm. Therefore, only solid part with diameter more than 10 mm was measured to calculate the ratio (*R* = (MD_S1_ + … + MD_S_ *_n_*)/MD_T_). It was classified into solid type when *R* > 60% and cystic type when *R* ≤ 60%. Some examples with the criterion were illustrated in detail (except for the missing mass with many solid components (*n* > 5). In Example 1 (case 11), the thickness of multiple regular septa was less than 5 mm and classified into cystic type. In Example 2 (case 5), the MD of the mass (solid line) was 75 mm, and the longest diameter of the solid part (dotted line) was 18.4 mm. So, the ratio is 24.5% (18.4/75) and categorized into cystic type. In Example 3 (case 12), the MD of the mass (solid line) was 67 mm, and the sum of the longest diameter of some solid parts (dotted line) was 38.9 mm (28.7 mm added to 10.1 mm). Thus, the ratio was 57.9% (38.9/67) and classified into cystic type. In Example 4 (case 16), the lesion had huge solid components beyond 60% and belonged to the solid type

A threshold value of 25% (proportion of solid components in a cyst‐solid tumor) is used in the Bosniak classification (version 2019) of renal cystic masses with solid components.^21^, ^22^ The Bosniak classification can be used for patients with cyst‐solid mass when the proportion of solid part ≤25%. But, the Bosniak classification cannot be applied when the percentage of the solid element >25%. However, it is difficult for radiologists to estimate qualitatively the volume percentage of the solid part on MDCT. The volume of mass and solid components are relative to their respective diameter (*D*) (*V* = (4/3)π*R* ^3^ and *R* = *D*/2). So, the MDs were used to replace the corresponding volume in our study, and the equation is as follow:

(1) $$\mathrm{Ratio}=MD_{\mathrm{solid}}/MD_{\mathrm{tumor}}$$

where MD_tumor_ is the maximal diameter of tumor in the slice in which the mass has the greatest size and MD_solid_ is the maximal diameter of the solid part in the same slice. When the ratio of the MD of the solid part in mass ≤60%, it was classified into cystic type (can be classified with Bosniak classification), and into solid type when the ratio >60% (cannot be classified with Bosniak classification). The detailed illustration is shown in Figure 1.

For lesions classified into cystic type, the number of septa, septal thickness, septal enhancement, wall thickness, wall enhancement, and mural nodularity on MDCT were assessed, and Table 1 gives the reference standard during image analysis. Lesions were classified into I–IV category with Bosniak classification.^21^

**TABLE 1:** The standard classification of mixed epithelial and stromal tumor of the kidney (MESTK) lesions with multidetector computed tomography (MDCT)

Solid type: unsuitable for the application of Bosniak classification (version 2019)	
Cystic type: can be classified with Bosniak classification (version 2019)	
I	Well‐defined cystic mass with thin (≤2 mm) smooth wall; homogeneous fluid (‐9 to 20 HU); no septa or calcifications; wall may enhance
II	Six types, all well‐defined with thin (≤2 mm) smooth walls: Cystic masses with thin (≤2 mm) and few (1–3) septa; septa and wall may enhance; may have calcification of any type^a^ Homogeneous hyperattenuating (>70 HU) masses at unenhanced MDCT Homogeneous non‐enhancing masses>20 HU at renal mass protocol MDCT, may have calcification of any type^b^ Homogeneous masses ‐9 to 20 HU at unenhanced MDCT Homogeneous masses 21–30 HU at portal venous phase MDCT Homogeneous low‐attenuation masses that are too small to characterize
IIF	Three types, cystic masses with enhancing wall or enhancing septa: Cystic masses with a smooth minimally thickened (3 mm) enhancing wall Cystic masses smooth minimal thickening (3 mm) of one or more enhancing septa Cystic masses with many (≥4) smooth thin (≤2 mm) enhancing septa
III	One or more enhancing thick (≥4 mm width) or enhancing irregular (displaying ≤3‐mm obtusely margined convex protrusion(s)) walls or septa
IV	One or more enhancing nodule(s) (≥4 mm convex protrusion with obtuse margins, or a convex protrusion of any size that has acute margins)

^a^ Renal masses have abundant thick or nodular calcifications on MDCT.

^b^ Renal tumors are hyperattenuating, homogeneous, non‐enhancing, and larger than 3 cm on MDCT.

### 2.4 Statistical analysis

An independent‐sample *t*‐test was used to assess differences in the MD, age, and R.E.N.A.L.‐NS between solid type and cystic type. The Fisher exact test was used to analyze gender, clinical symptoms, surgery methods, shape, SFI, calcification, septa, mural nodules, capsules, and enhancement patterns in the two groups. Statistical analyses were performed with SPSS 18.0 (SPSS Inc., Chicago, IL, USA). *p*‐Value > 0.05 was considered not statistically significant.

## 3 RESULTS

### 3.1 Clinical findings

**FIGURE 2:**
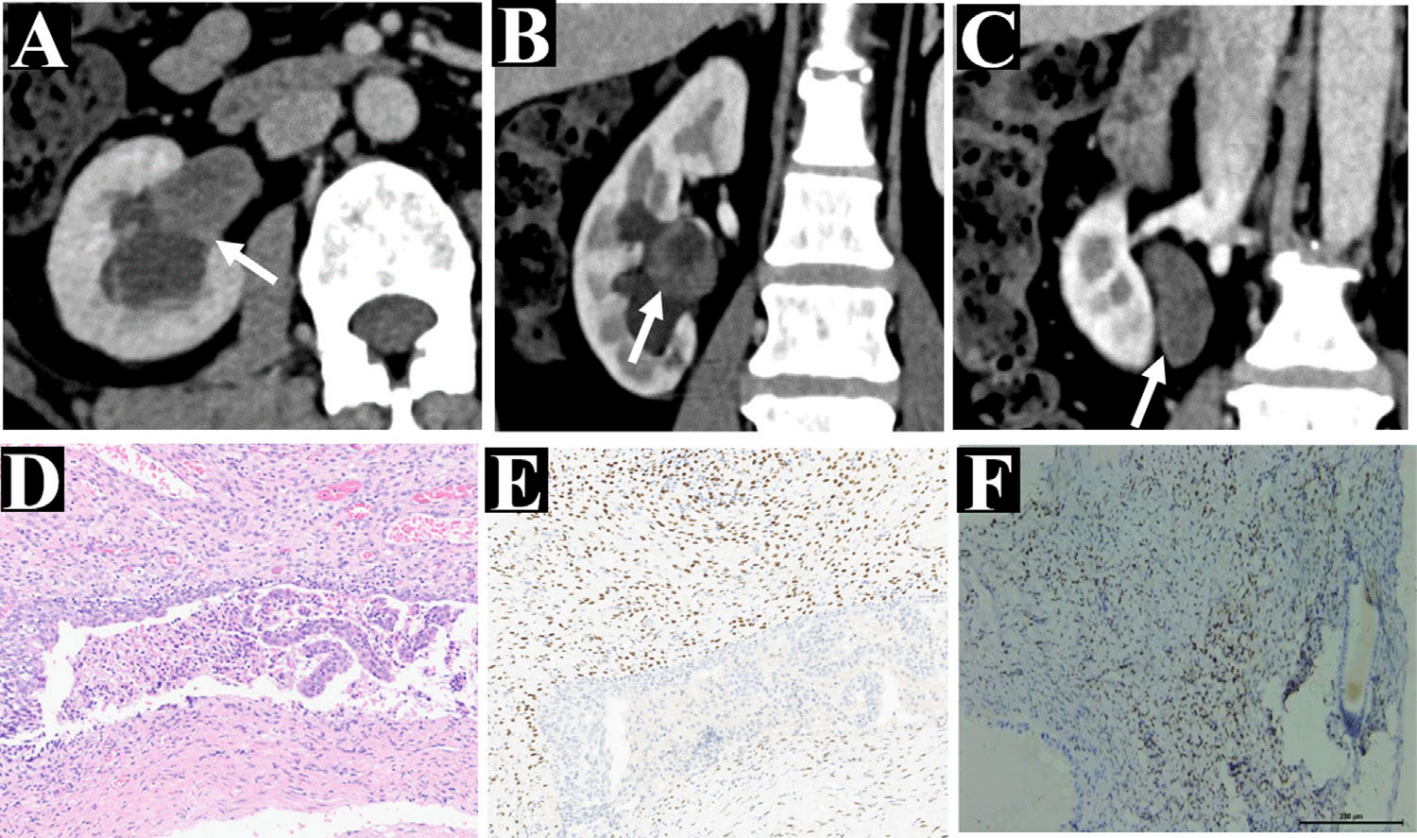
A 56‐year‐old woman with confirmed mixed epithelial and stromal tumor of the kidney (MESTK) by pathological examination of surgical specimen (case 3). Axial (a) and coronal (b and c) multidetector computed tomography (MDCT) images in the excretory phase. The images show the solid‐predominant mass extending into the pelvis and ureter (white arrow), which was confirmed histologically, (d) histopathological staining: consisted of the epithelium and stroma in the microscopy, (e) immunohistochemical staining: positive for estrogen receptors, represents the stromal element, and (f) immunohistochemical staining: progesterone receptor‐positive, illustrates the stromal cells

In the 17 patients, MESTK was detected incidentally in the majority of the patients without any symptoms (*n* = 10, 58.82%); five patients presented with flank pain and two patients presented with hematuria. MESTK occurred mainly in female (*n* = 13, 76.5%) compared to men (*n* = 4, 23.5%), with a predilection for perimenopausal women (*n* = 9, 69.23%). For R.E.N.A.L.‐NS, the score ranged between 7 and 11 with a mean score of 9.35 ± 1.17 (low: *n* = 0; moderate: *n* = 7 (41.18%); high: *n* = 10 (58.82%)) in our study. Two cases (11.76%) showed solid components extending into the renal pelvis and ureter (case . 3 and 16; Figure 2), and both of their R.E.N.A.L.‐NS were >10. All subjects underwent surgery (PN: *n* = 8 (47.06%); RN: *n* = 9 (52.94%)), and the longest follow‐up time was 114 months. No metastasis or recurrence was found in the patients after surgery and the renal function remained normal during the follow‐up period (an additional movie file shows this in more detail (see Additional Video)). The clinical details are shown in Table 2.

**TABLE 2:** Clinical features of the 17 patients with mixed epithelial and stromal tumor of the kidney (MESTK)

							R.E.N.A.L.‐NS				
Case no.	Follow‐up (months), begin and end time	Gender/age (years)	Symptoms	Menstrual status	Surgery	Creatinine level, before/after surgery (μmol/l)	R	E	N	L	Total score
1	2 (Jul 2020–Sep 2020)	M/26	Incidental	N	PN	85.7/107.0	3	2	3	1	9
2	6 (Mar 2019–Sep 2019)	M/52	Incidental	N	PN	96.0/118.0	3	3	1	1	8
3	9 (Feb 2019–Jun 2020)	F/56	Flank pain	Post‐	RN	59.0/53.0	2	3	3	3	11
4	16 (Jul 2018–Dec 2019)	F/45	Incidental	Peri‐	RN	88.7/98.3	3	2	3	2	10
5	24 (Feb 2018–Feb 2020)	F/53	Incidental	Post‐	RN	72.2/102.0	3	2	3	2	10
6	29 (Jun 2017–Nov 2019)	F/53	Incidental	Post‐	PN	82.0/69.0	1	3	1	2	7
7	30 (Jun 2017–Dec 2019)	F/49	Incidental	Peri‐	RN	64.2/106.2	3	2	3	3	11
8	30 (Jun 2017–Dec 2019)	F/34	Hematuria	Pre‐	RN	61.5/73.2	1	3	3	3	10
9	31 (Aug 2017–Mar 2020)	M/35	Incidental	N	PN	58.0/74.3	1	3	1	3	8
10	35 (Nov 2016–Oct 2019)	F/42	Incidental	Peri‐	PN	67.0/90.0	1	3	1	3	8
11	39 (Sep 2016–Nov 2019)	F/44	Hematuria	Peri	RN	99.0/131.0	2	2	3	3	10
12	43 (Sep 2016–Mar 2020)	M/61	Flank pain	N	RN	109.7/130.0	2	2	3	3	10
13	45 (Feb 2015–Nov 2018)	F/52	Incidental	Peri	PN	54.0/88.3	1	3	1	3	8
14	50 (Jul 2014–Sep 2018)	F/43	Incidental	Peri‐	RN	73.0/108.0	2	2	3	3	10
15	59 (Jun 2014–May 2019)	F/49	Flank pain	Peri‐	RN	78.4/96.3	1	3	3	3	10
16	60 (May 2013–May 2018)	F/52	Flank pain	Peri‐	PN	77.0/90.3	1	3	3	3	10
17	114 (May 2011–Nov 2020)	F/21	Flank pain	Pre‐	PN	59.6/68.5	3	1	2	3	9

*Note*: The number in the parentheses is the beginning and endpoint of follow‐up time; the creatinine level was normal from 53 to 132.6 μmol/L. The score standard for the evaluation of R.E.N.A.L.‐NS as follow: 1 point (R ≤ 40 mm; E ≥ 50%; N > 7 mm; or L: entirely above the upper or below the lower polar line); 2 points (40 < R < 70 mm; E < 50%; 4 mm < N < 7 mm; or L: lesion crosses polar line); 3 points (R ≥ 70 mm; E: entirely endophytic; N ≤ 4 mm; L: >50% of mass is across polar line or mass crosses the axial renal midline or mass is entirely between the polar lines).^13^

Abbreviations: peri‐, perimenopausal; PN, partial nephrectomy; post‐, postmenopausal; pre‐, premenopausal; R.E.N.A.L.‐NS, R.E.N.A.L.‐ nephrometry; RN, radical nephrectomy.

### 3.2 Imaging findings

**FIGURE 3:**
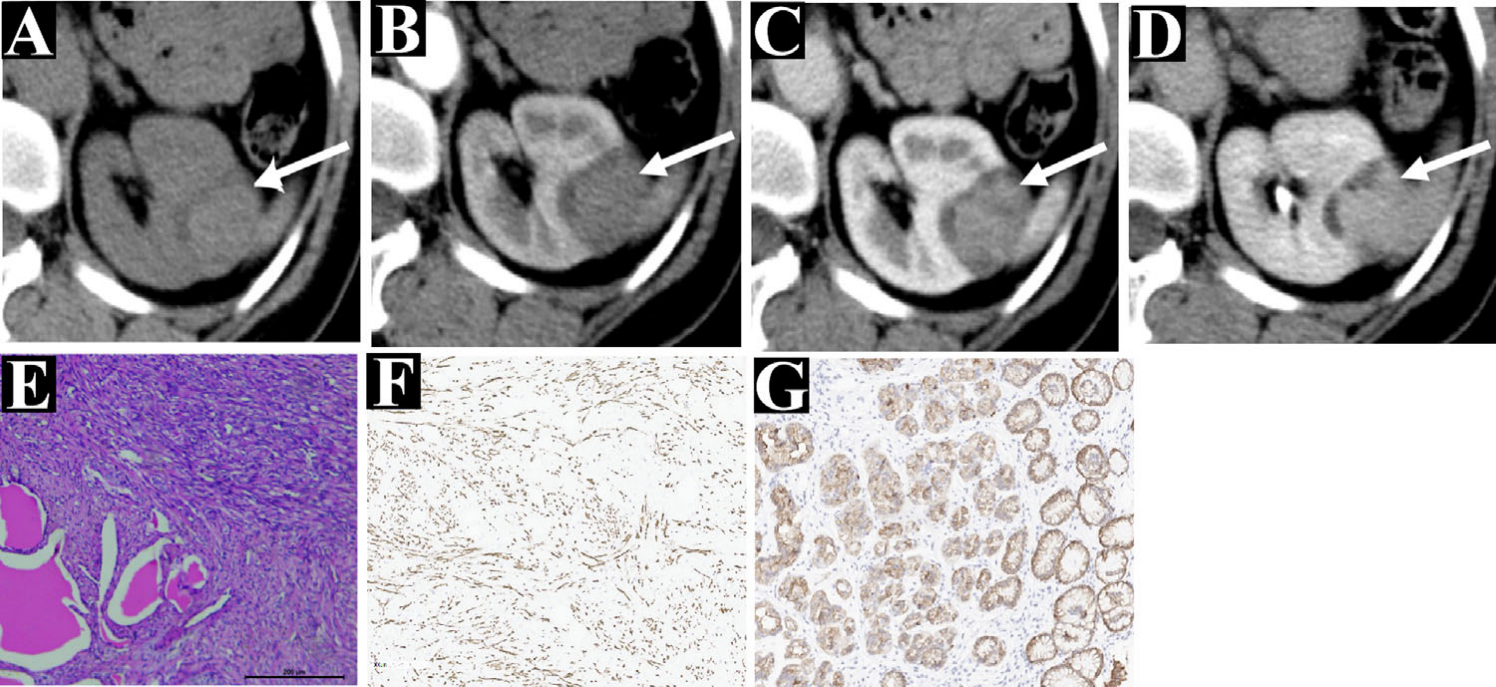
A 52‐year‐old woman (case 13). A slightly hyperdense solid mass in the left kidney was found accidentally on the axial unenhanced multidetector computed tomography (MDCT) (a), which shows no enhancement on the corticomedullary phase (b), slight enhancement on the nephrographic phase (c), further enhancement on the excretion phase (d). It was confirmed as mixed epithelial and stromal tumor of the kidney (MESTK) by histopathological staining with the epithelium and stroma (e) and immunohistochemical staining ((f) positive for smooth muscle actin, representing the stromal area; (g) positive for pan‐cytokeratin, representing epithelial areas). The lesion (case 13) was classified into solid type according to the equation

**FIGURE 4:**
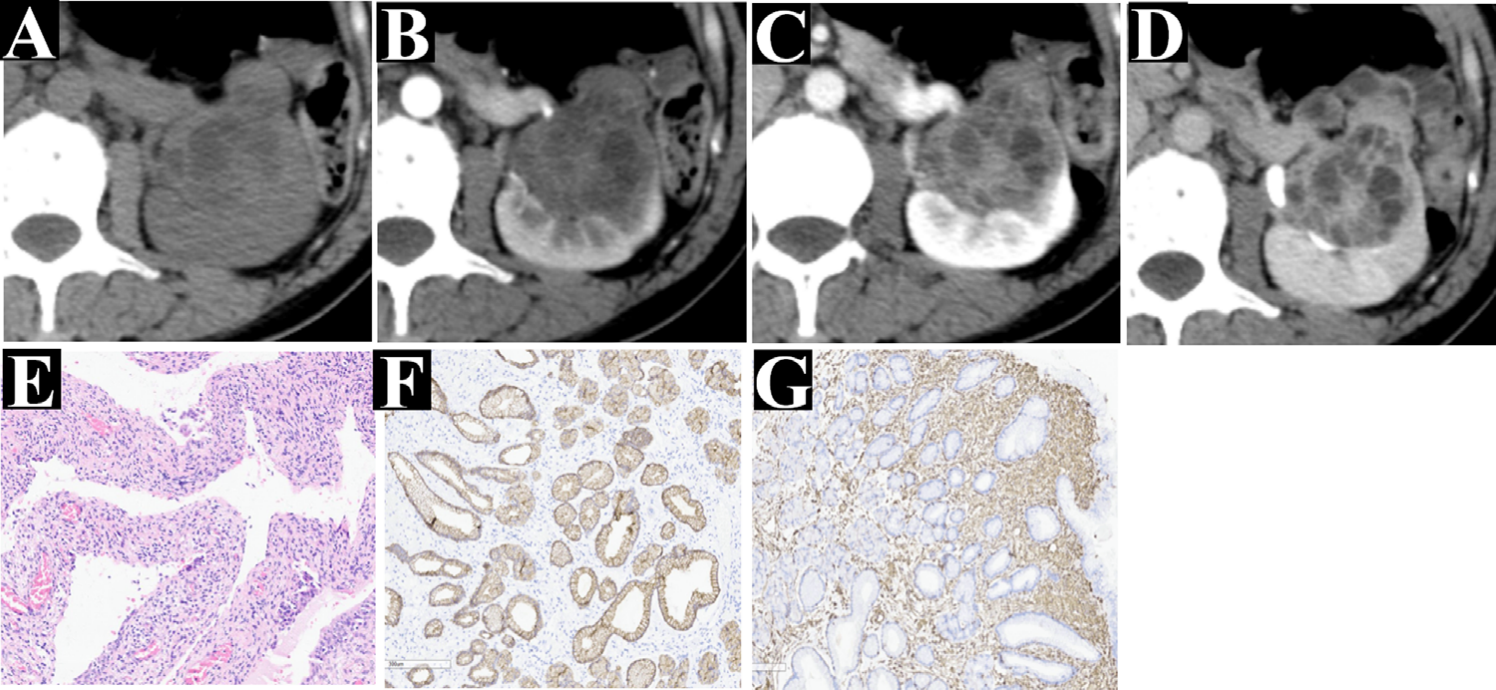
A 49‐year‐old woman with flank pain (case 15). A cystic‐solid mass is shown on an axial enhancement multidetector computed tomography (MDCT) (a), which displayed slight enhancement with multi‐septate cystic and solid components on the corticomedullary phase (b), prolonged enhancement on the nephrographic phase (c) and the excretory phase (d), and was confirmed as mixed epithelial and stromal tumor of the kidney (MESTK) by histopathological (e) and immunohistochemical ((f) the positive for pan‐cytokeratin in the epithelium; (g) positive for vimentin in the stromal area). The patient (case 15) was classified into cystic type (IV category). Compared with Figure 3 (case 13), it has a variable proportion of cystic and solid components

**FIGURE 5:**
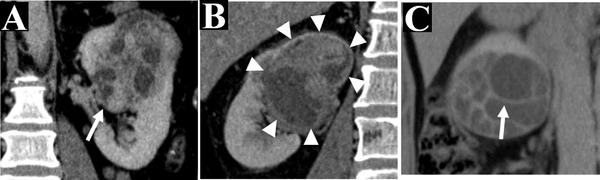
Some vital imaging features on multidetector computed tomography (MDCT). Renal sinus fat invagination (SFI) (white arrow) is shown on the sagittal MDCT (a, case 7), which represents the tumor invasion into the sinus fat (white arrow). In addition, the capsule of the tumor (white triangle) is shown on a coronal MDCT (b, case 12), and the multiple regular septa (white arrow) is shown in the tumor in the coronal MDCT (c, case 11)

All MESTKs demonstrated a variable proportion of multi‐septate cystic and solid components, which appeared as delayed enhancement with a well‐circumscribed margin on triphasic dynamic‐enhanced MDCT (Figures 3 and 4), except for two subjects that had gradual washout pattern. The CT attenuation was less than 20 HU in the cystic part. In the 17 cases, the tumor in 14 patients (77.8%) showed a regular shape, and 10 patients (58.82%) demonstrated SFI (Figure 5a). In addition, tumor capsule was present in seven patients (41.1%) (Figure 5b), and regular or irregular septa was present in four patients (22.2%) (Figure 5c). The MDCT findings are shown in Table 3.

**TABLE 3:** Multidetector computed tomography (MDCT)^a^ imaging features of the 17 patients with mixed epithelial and stromal tumor of the kidney (MESTK)

Case no.	Type	BC	MD (mm)	Shape	Location	SFI	Calcification	Septa	Mural nodule	Capsule	Enhancement pattern
1	ST	NA	75	Re‐	R‐1	N	N	N	N	N	GE
2	ST	NA	70	Re‐	L‐1	N	Y	N	N	N	GE
3	ST	NA	43	Re‐	R‐3	Y	N	N	N	N	GE
4	CT	III	112	Irre‐	R‐2	Y	N	Re‐	N	Y	GE
5	CT	III	75	Re‐	L‐2	Y	Y	Re‐	N	Y	GE
6	ST	NA	21	Re‐	R‐2	N	N	N	N	N	GW
7	CT	IV	109	Irre‐	L‐3	Y	N	Irre‐	Y	Y	GW
8	CT	IV	39	Re‐	L‐3	Y	N	Irre‐	N	N	GE
9	ST	NA	8	Re‐	L‐3	N	N	N	N	N	GE
10	ST	NA	27	Re‐	L‐3	N	N	N	N	N	GE
11	CT	III	64	Re‐	R‐3	Y	N	Re‐	N	Y	GE
12	CT	IV	67	Irre‐	R‐3	Y	Y	Irre‐	Y	Y	GE
13	ST	NA	29	Re‐	L‐3	N	N	N	N	N	GE
14	ST	NA	68	Re‐	R‐3	Y	N	N	N	N	GE
15	CT	IV	37	Re‐	L‐3	Y	N	Irre‐	N	Y	GE
16	ST	NA	30	Re‐	R‐3	Y	Y	N	N	N	GE
17	CT	II	145	Re‐	L‐3	N	N	Re‐	N	Y	GE

*Note*: L: left; R: right; 1: entirely above the upper or below the lower polar line; 2: lesion crosses polar line; 3: >50% of mass is across polar line or mass crosses the axial renal midline or mass is entirely between the polar lines.^13^

Abbreviations: BC, Bosniak classification; CT, cystic type; GE, gradual enhancement; GW, gradual washout; Irre‐, irregular; MD, maximal diameter of tumor; NA, not applicable; Re‐, regular; SFI, sinus fat invagination; ST, solid type.

^a^ The scanning parameters: slice thickness of 1 mm, slice gap of 0 mm, the pitch of 1.2, 100 kVp and 200 mA for Somatom Definition and Aquilion ONE.

### 3.3 The comparison of solid type with cystic type

In our study, nine patients with MESTK were classified into solid type (Figure 3) and eight patients into cystic type (Figure 4). Based on the Bosniak classification, the lesions in the cystic type group were classified as category I (*n* = 0), II (*n* = 0), II F (*n* = 1), III (*n* = 3), and III (*n* = 4). Compared with solid type, the lesions in cystic type have larger MD (81.00 ± 37.91 vs. 41.22 ± 24.19, *p* = 0.020), higher R.E.N.A.L.‐NS (10.03 ± 0.50 vs. 8.95 ± 1.26, *p* < 0.001), higher RN (75.00% vs. 22.22%, *p* = 0.015), greater SFI (87.5% vs. 33.3%, *p* = 0.05), septa (100% vs. 0%, *p* < 0.001), and more capsule (100% vs. 11.1%, *p* < 0.001). Besides, there was no significant difference between the solid type and cystic type groups regarding age, gender, clinical symptoms, the shape of the tumor, location of the tumor, calcification, mural nodule, and enhancement pattern (all *p*‐values > 0.05) (Table 4).

**TABLE 4:** The comparison between two types of mixed epithelial and stromal tumor of the kidney (MESTK)

	Solid type (*n* = 9)	Cystic type (*n* = 8)	*p*‐Value
Clinical features			
Age (years)	45.67 ± 10.01	44.50 ± 12.24	0.834
MD (mm)	41.22 ± 24.19	81.00 ± 37.91	0.020*
R.E.N.A.L.‐NS	8.95 ± 1.26	10.03 ± 0.50	<0.001*
Gender			0.576
F	6 (66.67%)	7 (87.50%)	
M	3 (33.33%)	1 (12.50%)	
Clinical symptoms			0.347
Y	3 (33.33%)	5 (62.50%)	
N	6 (66.67%)	3 (37.50%)	
Surgery methods			0.015*
PN	7 (77.78%)	1 (25.00%)	
RN	2 (22.22%)	7 (75.00%)	
MDCT features			
Shape			0.082
Re‐	9 (100.00%)	5 (62.50%)	
Irre‐	0 (0.00%)	3 (37.50%)	
Location			0.637
L	4 (44.44%)	5 (62.50%)	
R	5 (55.56%)	3 (37.50%)	
SFI			0.050*
Y	3 (33.33%)	7 (87.50%)	
N	6 (66.67%)	1 (12.50%)	
Calcification			1.000
Y	2 (22.22%)	2 (25.00%)	
N	7 (77.78%)	6 (75.00%)	
Septa			<0.001*
Y	0 (0.00%)	8 (100.00%)	
N	9 (100.00%)	0 (0.00%)	
Mural nodule			0.206
Y	0 (0.00%)	2 (25.00%)	
N	9 (100.00%)	6 (75.00%)	
Capsule			<0.001*
Y	0 (0.00%)	7 (87.50%)	
N	9 (100.00%)	1 (12.50%)	
Enhancement pattern			0.131
Het‐	8 (89.89%)	4 (50.00%)	
Hom‐	1 (11.11%)	4 (50.00%)	

Abbreviations: F, female; Het‐, heterogeneous; Hom‐, homogeneous; Irre‐, irregular; L, left; M, male; MD, maximal diameter of tumor; N, no; PN, partial nephrectomy; Re‐, regular; R.E.N.A.L.‐NS, R.E.N.A.L.‐nephrometry score; R, right; RN, radical nephrectomy; SFI, sinus fat invagination; Y, yes.

* *p*‐Value not beyond 0.050.

## 4 DISCUSSION

As a member of the mixed epithelial and stromal tumor family, the imaging features of MESTK and its connection with patients’ management remain largely unknown.^5^ Most of the current literature on MESTK focused on its pathologic features,^23^, ^24^ with only a small number of case reports and case series addressing its radiologic features.^12^, ^25^ In our study, 17 patients with MESTK who had an MDCT were collected to analyze the imaging and clinical features and with emphasis on providing a reference for patients’ management based on the difference in the two types of MESTK.

The typical clinical symptomatology of MESTK includes flank pain, hematuria, or symptoms related to genitourinary infections.^23^ In our research, only five patients with flank pain and two patients with hematuria were found in the 17 MESTK cases. However, most of them (10 patients, 58.82%) were detected MESTK incidentally without any symptoms, which was consistent with the findings of Lane et al.^15^ The possible reason may be advances in imaging modalities and the prevalence of health examinations. Also, MESTKs were found in 13 females (76.5%): nine of whom were perimenopausal (69.23%). This suggests that perimenopausal females had the predominance of MESTK due to the serum estrogen level. The findings are in accordance with previous studies.^23^, ^26^, ^27^

R.E.N.A.L.‐NS is a crucial scoring system for patients with renal masses, promoting a standardized communication and standardization of clinical care patterns.^13^ Renal tumors with low to moderate complexity (R.E.N.A.L.‐NS ≤ 9 scores) are often treated with PN, whereas RN is done for high complex lesions (R.E.N.A.L.‐NS > 9 scores).^28^ In our study, the range of R.E.N.A.L.‐NS was 7–11 score and the mean score was 9.35 ± 1. 17 score (low: *n* = 0; moderate: *n* = 7 (41.18%); high: *n* = 10 (58.82%)). Seven patients (R.E.N.A.L.‐NS ≤ 9 score) underwent PN and nine patients (R.E.N.A.L.‐NS > 9 score) had RN. Only one patient (case 16, R.E.N.A.L.‐NS = 10 score) chose PN due to the patient's strong desire to preserve the kidney and because the tumor was located mainly in the pelvis and ureter. Also, there were no signs of recurrence or metastasis for all patients during the follow‐up time, although the metastasis of MESTK has been reported.^29^ It indicated that the choice of treatment is successful for all persons. However, R.E.N.A.L.‐NS score includes the evaluation of radius (R) for the tumor size as MD, exophytic/endophytic properties of the tumor (E), nearness of tumor deepest portion to the collecting system or sinus (N), anterior/posterior descriptor (A), and the location relative to the polar line (L),^13^ which is complex for those patients with MESTK. Comparison with R.E.N.A.L.‐NS, the classification (solid and cystic type) is easier to evaluate only by the proportion of solid components.

MESTKs are unilateral and single lesions with different proportions of solid components.^10^, ^12^, ^13^, ^19^ Our cases demonstrated well‐circumscribed expansile lesions with a variable proportion of multi‐septate cystic and solid components, having delayed enhancement on triphasic dynamic‐enhanced MDCT. The delayed enhancement may be related to the abundance of collagen fibers, which restrict the diffusion of contrast agent within the tumor.^4^, ^20^ Lane et al.^15^ found that MESTK commonly tends to extend into the renal sinus. In our study, 10 subjects (55.5%) have demonstrated SFI, indicating that nephron‐sparing surgery is not the best management plan for such tumors compared to those without the SFI.

In our study, solid type in MESTK is composed mainly of solid elements (>60% for the ratio of the solid components’ MD in the mass), while cystic type consists principally of the cystic part (≤60% for the percentage of the solid element’ MD in the tumor). Interestingly, the MD of tumor in cystic type was greater than solid type. The reason may be that epithelial elements existed more in cystic type than solid type. These epithelia then secret fluid that accumulates in many various cysts due to the epithelial cells containing much spatulate papillary architectures and tiny crowded glands.^4^ In addition, our findings showed that R.E.N.A.L‐NS in cystic type was higher than solid type (10.03 ± 0.50 vs. 8.95 ± 1.26, *p* < 0.001), which implies that patients in cystic type had a higher risk for urine leakage when PN is performed.^30^ Fortunately, the patients with cystic type underwent RN and therefore had no urine leakage.

SFI is not currently used in either the Robson or TNM staging systems. However, it is an important prognostic finding and staging parameter.^17^ Bertini et al.^18^ deemed that SFI significantly influenced disease‐free survival and cancer‐specific survival in patients without nodal or distant metastases. Bonsib et al.^17^ suggested that RN should be recommended for those patients with renal sinus invasion. It is in line with our findings that seven out of eight patients (cystic type) with SFI had RN. Furthermore, cystic type had more septa than solid type and met the Bosniak classification requirement, but solid type did not satisfy it. Thus, MESTK cystic type in our study presents as a multiloculated lesion with regular or irregular septa, which is different from solid type.^14^ In addition, cystic type lesions have more capsules, caused by compression of adjacent renal tissue by the tumors correlating with the larger size of these tumors.^31^, ^32^

Limitations of this study are the small number of MESTK cases, retrospective design, and single‐center study. With advances in imaging modalities and the rising popularity of health examination, diagnosis of asymptomatic and small MESTK will increase and promote a better treatment plan for patients’ management.

## 5 CONCLUSION

In general, MESTK presents as a well‐circumscribed lesion with a variable proportion of multi‐septate cystic and solid components, showing a delayed enhancement on triphasic dynamic‐enhanced MDCT. With a comparison of solid type, cystic type MESTK has more hazardous features, such as larger MD, higher R.E.N.A.L.‐NS, more RN, greater SFI, multiple septa, which suggests that RN is more suitable for cystic type lesions. Nephron‐sparing surgery should be considered for solid type cases.

## AUTHOR CONTRIBUTIONS

Juan Chen, Hui Liu, Yigang Pei, and Wenzheng Li conceived and designed the study. Juan Chen, Mengsi Li, and Hui Liu collected and analyzed the data. Wenguang Liu and Ismail Bilal Masokano contributed analysis tools. Juan Chen, Hui Liu, Yigang Pei, and Wenzheng Li provided critical inputs on design, analysis, and interpretation of the study. All the authors had access to the data. All authors read and approved the final manuscript as submitted.

## ETHICAL APPROVAL

Ethical approval was obtained from Institutional Review Board of the Xiangya Hospital, Central South University. This retrospective study was performed in accordance with the provisions of the Declaration of Helsinki. The requirement for informed consent was exempted by the Institutional Review Board of the Xiangya Hospital, Central South University as all data were analyzed anonymously (Approved number 2018111101).

## CONFLICT OF INTEREST

The authors declare no conflict of interest.

## Supporting information

SUPPORTING INFORMATION

SUPPLEMENTAL VIDEO 1

SUPPLEMENTAL MOVIE 1

## Data Availability

Research data are not shared.
