# Supplementary material for: Differentiating the clinical and computed tomography imaging features of mixed epithelial and stromal tumors of the kidney to establish a treatment plan
Source: J Appl Clin Med Phys. 2021 Dec 3;23(1):e13486. doi: 10.1002/acm2.13486 (PMC8803287; doi:10.1002/acm2.13486)
Supplement: Supplementary file 1 — SUPPORTING INFORMATION [file ACM2-23-e13486-s001.doc]

**Differentiating the clinical and computed tomography imaging features of mixed epithelial and stromal tumors of the kidney to establish a treatment plan**

Juan Chen^#1^, Hui Liu^#1^, Mengsi Li^1^, Wenguang Liu^1^, Ismail Bilal Masokano^1^, Yigang Pei*^1,2^ ，Wenzheng Li*^1^

^1^ Department of Radiology, Xiangya Hospital, Central South University, Changsha, 410008, China.

^2^ Postdoctoral Fellow, Xiangya Hospital, Central South University, Changsha, Hunan, 410008, P.R. China.

**The corresponding authors(*) are Wenzheng Li and Yigang Pei .**

**Name:** Wenzheng Li, M.D

**Address:** Department of Radiology, Xiangya Hospital, Central South University, Changsha, 410008, China

**The telephone number:** +0086-13574853656

**Fax number:** +0086-0731-89753812,

**The e-mail address:** **wenzheng727@163.com**

**Name:** Yigang Pei，M.D

**Address:** Department of Radiology, Xiangya Hospital, Central South University, Changsha, 410008, China

**The telephone number:** +0086-15388927005

**Fax number:** +0086-0731-89753812,

**The e-mail address:** **xypyg0731@163.com**

**The first authors (#) are Juan Chen and Hui Liu.**

**Name: Juan Chen**

**The e-mail address:** **cj05251012@163.com**

**Name: Hui Liu**

**The e-mail address: yx_liuhui@163.com**

**Posted history:** This manuscript was pre-printed to Reserch Square:doi: https://doi.org/10.21203/rs.3.rs-350581/v1

**Authors' contributions**: Juan Chen, Hui Liu, Yigang Pei and Wenzheng Li conceived and designed the study. Juan Chen, Mengsi Li and Hui Liu collected and analyzed the data. Wenguang Liu and Ismail Bilal Masokano contributed analysis tools. Juan Chen, Hui Liu, Yigang Pei and Wenzheng Li provided critical inputs on design, analysis, and interpretation of the study. All the authors had access to the data. All authors read and approved the final manuscript as submitted.

**Ethics approval**: Ethical approval was obtained from Institutional Review Board of the Xiangya Hospital, Central South University. This retrospective study was performed in accordance with the provisions of the Declaration of Helsinki. The requirement for informed consent was exempted by the Institutional Review Board of the Xiangya Hospital, Central South University as all data was analyzed anonymously (Approved number 2018111101).
